# Physical inactivity as a risk factor for all-cause mortality in Brazil (1990–2017)

**DOI:** 10.1186/s12963-020-00214-3

**Published:** 2020-09-30

**Authors:** Diego Augusto Santos Silva, Mark Stephen Tremblay, Fatima Marinho, Antonio Luiz Pinho Ribeiro, Ewerton Cousin, Bruno Ramos Nascimento, Paulo da Fonseca Valença Neto, Mohsen Naghavi, Deborah Carvalho Malta

**Affiliations:** 1 Physical Education Department, Research Center in Kinanthropometry and Human Performance, Federal University of Santa Catarina, Trindade, Florianopolis, SC 88040-900 Brazil; 2 Children’s Hospital of Eastern Ontario Research Institute, Ottawa, ON Canada; 3 Department of Health Surveillance, Ministry of Health, Brasília, DF Brazil; 4 Federal University of Minas Gerais, Belo Horizonte, MG Brazil; 5 Graduate Program of Epidemiology, Federal University of Rio Grande do Sul, Porto Alegre, RS Brazil; 6 Clinical Hospital of the Federal University of Minas Gerais, Belo Horizonte, MG Brazil; 7 Institute for Health Metrics and Evaluation, Seattle, WA USA

**Keywords:** Adults, Epidemiology, Disease burden, Physical activity, Morbidity

## Abstract

**Background:**

The aim of this study was to estimate the mortality from all causes as a result of physical inactivity in Brazil and in Brazilian states over 28 years (1990–2017).

**Methods:**

Data from the Global Burden of Disease (GBD) study for Brazil and states were used. The metrics used were the summary exposure value (SEV), the number of deaths, age-standardized mortality rates, and the fraction of population risk attributable to physical inactivity.

**Results:**

The Brazilian population presented risk of exposure to physical inactivity of (age-standardized SEV) of 59% (95% U.I. 22–97) in 1990 and 59% in 2017 (95% U.I. 25–99). Physical inactivity contributed a significant number of deaths (1990, 22,537, 95% U.I. 12,157–34,745; 2017, 32,410, 95% U.I. 17,976–49,657) in the analyzed period. These values represented mortality rates standardized by age (per 100,000 inhabitants) of 31 (95% U.I. 17–48) in 1990 and 15 (95% U.I. 8–23) in 2017. From 1990 to 2017, a decrease in standardized death rate from all causes attributable to physical inactivity was observed in Brazil (− 52%, 95% U.I. − 54 to − 49). The Brazilian states with better socioeconomic conditions presented greater reductions in age-standardized mortality (male: *rho* = 0.80; female: *rho* 0.84) over the period of 28 years.

**Conclusions:**

These findings support the promotion of physical activity in the Brazilian population for the prevention of early mortality.

## Background

Physical inactivity is associated with the early onset of noncommunicable chronic diseases that lead to health problems and to all-cause mortality, regardless of other risk factors [1–3]. This relationship has been researched since the 1950s [4] and makes physical inactivity one of the main modifiable health risk factors in all age groups.

Data from the Global Burden of Disease (GBD) study revealed that physical inactivity was risk factor accounting for approximately 1.3 million deaths (17 deaths per 100,000 inhabitants) in individuals aged 25 years and over [5]. These alarming data combined with a number of systematic reviews and meta-analyses that highlighted physical inactivity as a pandemic [6, 7] supported the publication of the World Health Organization Global Plan of Action for Physical Activity 2018–2030 [8]. This document aims to provoke a relative reduction in physical inactivity of 10% by 2025 and of 15% by 2030 [8], which will contribute to a longer life expectancy of the population [1, 8].

The estimate of the association between physical inactivity and all-cause mortality among the Brazilian population is not known. To date, what has been reported for Brazil are estimates of physical inactivity and all-cause mortality from a single year [9] and from a single city in the country [10]. This information is important but does not allow the analyses of trend indicators of all-cause mortality as a result of physical inactivity. Thus, the present study will contribute new, important, and timely evidence related to this subject.

The present study aims to analyze the temporal evolution (1990 to 2017) of mortality rates for all causes attributable to physical inactivity in Brazil and in Brazilian states.

## Methods

### General aspects

An analytical study based on estimates of global burden of disease for Brazil made by GBD 2017 was carried out, coordinated by the Institute for Health Metrics and Evaluation (IHME) in partnership with the Ministry of Health of Brazil [11–15]. In the analysis of mortality, information from the Mortality Information System of the Brazilian Ministry of Health was used, with adjustment for underreporting of deaths and declaration of undefined/nonspecific causes, called garbage codes [13–15].

The standardized methodology of analysis adopted by the GBD makes it possible to compare countries, regions, and subnational data, also enabling analyzing trends, provided that the time series data are adjusted and comparable [13–15].

### Mortality estimate due to diseases that have physical inactivity as risk factor

In the present study, physical inactivity was considered a risk factor for breast cancer, colorectal cancer, ischemic heart disease, diabetes mellitus, and stroke [5]. Other causes of mortality that have physical inactivity as one of the risk factors were not determined in the GBD study methodology. Information on the records and how each of these diseases was collected, estimated, and adjusted are found elsewhere in the literature [5, 13–15].

The comparative risk assessment conceptual framework used in GBD study established a causal web of hierarchically organized risks or causes that contribute to health outcomes, which allows for quantification of risks or causes at any level in the framework [5]. In GBD 2017, as in previous iterations of the GBD study, we evaluated a set of behavioral, environmental and occupational, and metabolic risks, where risk-outcome pairs were included based on evidence rules [5]. These risks were organized in four hierarchical levels, where level 1 represents the overarching categories (behavioral, environmental and occupational, and metabolic) nested within level 1 risks; level 2 contains both single risks and risk clusters; level 3 contains the disaggregated single risks from within level 2 risk clusters; and level 4 details risks with the most granular disaggregation. Physical inactivity is in the category of behavioral risk factor and stands out at hierarchical level 2 [5]. In addition, physical inactivity does not have any level of disaggregation.

### Physical inactivity prevalence estimate

Surveys of the general adult population performed using random sampling procedures that assessed self-reported physical activity in all life domains (leisure/recreation, work, household, and transport) were included. Studies that evaluated only one of the physical activity domains were not included [5].

In general, for GBD estimates, data are primarily derived from two questionnaires, the Global Physical Activity Questionnaire (GPAQ) and the International Physical Activity Questionnaire (IPAQ). However, other studies that evaluated physical activity intensity, frequency, and duration in all domains were included [5].

In the case of Brazil, surveys considered included the National Health Survey 2013, Surveillance System for Risk Factors and Protection for Chronic Diseases by Telephone Inquiry (VIGITEL), Household Survey on Risk Behaviors and Referred Morbidity of Noncommunicable Diseases 2002–2005, World Health Survey 2003, and the International Study on the Prevalence of Physical Activity. Further details can be found at http://ghdx.healthdata.org/gbd-2016/data-input-sources?locations=135&components=6&risks=125.

To standardize all physical inactivity estimates in Brazil, data from the population aged 25 years or more were considered. Physical activity was considered only when accumulated for at least 10 consecutive minutes or more. Physical activity frequency, duration, and intensity were used to calculate the total metabolic equivalent (MET) minutes in the week. Physical activity level was categorized by total MET-minutes per week. The lower limit (600 MET-min/week) is the recommended minimum amount of physical activity to get any health benefit [5, 16]. More details on these models can be found in the literature [5].

### Analysis strategy

The contribution of physical inactivity to mortality from all causes investigated in this study was estimated using a conceptual framework of the comparative risk factor assessment [5]. For this, the CODEm simulation model, which is an analytical tool that tests various statistical models of causes of death and creates a combination of models that provide the best predictive performance, was used to estimate indicators by sex, age, state, year, and cause. The DisMod-MR 2.1 software (World Health Organization©, Geneva, Switzerland), a meta-regression tool, was used to simultaneously derive estimates of prevalence, disability, and mortality due to physical inactivity [5]. Spatiotemporal Gaussian process regression (ST-GPR) has been used for risk factors where the data density is sufficient to estimate a very flexible time trend. The approach is a stochastic modelling technique that is designed to detect signals amidst noisy data. It also serves as a powerful tool for interpolating non-linear trends. Unlike classical linear models that assume that the trend underlying data follows a definitive functional form, GPR assumes that the specific trend of interest follows a Gaussian process. Details of all models can be found in literature [5].

In this study, the counterfactual level of risk exposure used is the risk exposure that is both theoretically possible and minimizes risk in the exposed population that consequently captures the maximum population attributable burden [5]. For each risk evaluated in GBD study, included low physical activity, has been used the best available epidemiological evidence from published and unpublished relative risks by level of exposure and the lowest observed level of exposure from cohorts used to select a single level of risk exposure combined to establish the theoretical minimum-risk exposure level (TMREL) [5]. The TMREL for physical activity is 3000–4500 MET-min per week, which was calculated as the exposure at which minimal deaths across outcomes occurred [5].

We also estimated the population attributable fraction (PAF), which represents the proportion of risk that would be reduced in a given year if the exposure to a risk factor in the past was reduced to an ideal exposure scenario [5]. We used a recently published dose-response meta-analysis of prospective cohort studies to estimate the effect size of the change in physical activity level on breast cancer, colon cancer, diabetes, ischemic heart disease, and ischemic stroke [3].

Summary exposure value (SEV) for physical inactivity was used in this study. SEV is the relative risk-weighted prevalence of exposure, a univariate measure of risk-weighted exposure, taking the value zero when no excess risk for a population exists and the value one when the population is at the highest level of risk. We report SEVs on a scale from 0 to 100% where a decline in SEV indicates reduced exposure to physical inactivity and an increase in SEV indicates increased exposure. More details on SEV are also available elsewhere [5, 13–15].

The absolute numbers of deaths and mortality rates (per 100,000 inhabitants, crude, and age-standardized) were also used as metrics [5, 13–15]. In addition, the 95% uncertainty intervals were estimated (95% U.I.).

Analyses were performed for the Brazilian population and also stratified by sex and age (25–49 years, 50–69 years, 70+ years). Analyses were presented for each of the Brazilian states plus the Federal District in the years of 1990 and 2017. The GBD study created the Socioeconomic Development Index (SDI) [13–15] for all evaluated locations, by calculating per capita income, formal education at 15 years of age, and fertility rate. This index was used to compare the metrics among Brazilian states. For this, the Spearman correlation coefficient was applied.

## Results

The Brazilian population presented risk of exposure to physical inactivity of (age-standardized SEV) 59% (95% U.I. 22–97) in 1990 and 59% in 2017 (95% U.I. 25–99). These values represented a stability of the Brazilian population in the exposure to physical inactivity over 28 years. All the Brazilian states presented this stability in the age-standardized SEV from 1990 to 2017 (data not shown in the tables/figures).

In Brazil, it was estimated that 22,537 (95% U.I. 12,157–34,745) deaths from all causes were attributable to physical inactivity in 1990. In 2017, 32,410 (95% U.I. 17,976–49,657) deaths were estimated from all causes attributable to physical inactivity. These values represented mortality rates standardized by age (per 100,000 inhabitants) of 31 (95% U.I. 17–48) in 1990 and 15 (95% U.I. 8–23) in 2017. From 1990 to 2017, a decrease in standardized death rate from all causes attributable to physical inactivity was observed in Brazil (− 52%, 95% U.I. − 54 to − 49). From 1990 to 2017, the Brazilian states with the highest percentage reductions in age-standardized mortality rate for all causes attributable to physical inactivity were Espírito Santo (− 64%, 95% U.I.: − 67 to − 60), Paraná (− 60%, 95% U.I. − 62 to − 57), and São Paulo (− 60%, 95% U.I. − 62 to − 57). Table 1 shows information on all-cause deaths attributable to physical inactivity per Brazilian state in 1990 and 2017.

**Table 1:** Number and age-standardized mortality rate for all-causes in Brazil in ages ≥ 25 years

	Mortality for all-causes^**†**^ due to physical inactivity														
	1990			2017			1990			2017			Change (1990-2017)		
	Deaths	95% U.I.		Deaths	95% U.I.		Rate*	95% U.I.		Rate*	95% U.I.		%*	95% U.I.	
**Brazil**	22,537	12,157	34,745	32,410	17,976	49,657	31	17	48	15	08	23	− 52	− 54	− 49
**Acre**	32	17	49	66	36	101	24	13	38	13	07	19	− 48	− 52	− 43
**Alagoas**	311	170	481	510	286	775	26	14	39	17	10	26	− 32	− 38	− 25
**Amapá**	17	09	27	51	29	77	24	13	36	13	07	19	− 46	− 49	− 42
**Amazonas**	136	72	209	284	159	433	22	12	35	12	07	19	− 46	− 50	− 40
**Bahia**	1383	753	2126	2163	1229	3284	22	12	34	14	08	21	− 38	− 42	− 32
**Ceará**	652	355	1016	1314	722	2021	17	09	26	13	07	20	− 21	− 29	− 12
**Distrito Federal**	118	64	181	241	136	369	31	17	47	14	08	21	− 55	− 58	− 52
**Espírito Santo**	360	193	559	523	292	794	36	19	56	13	07	20	− 64	− 67	− 60
**Goiás**	372	200	575	794	440	1221	28	15	43	13	07	20	− 53	− 56	− 49
**Maranhão**	406	221	631	845	466	1316	18	10	27	14	08	21	− 21	− 27	− 14
**Mato Grosso**	163	87	256	356	199	548	27	14	42	13	07	21	− 50	− 54	− 44
**Mato Grosso do Sul**	207	111	320	413	228	636	31	16	47	16	09	25	− 47	− 50	− 44
**Minas Gerais**	2349	1258	3640	3046	1677	4675	31	17	49	12	07	19	− 61	− 63	− 59
**Pará**	439	229	677	841	467	1286	25	13	39	14	08	20	− 44	− 49	− 38
**Paraíba**	479	266	731	791	431	1219	22	12	33	17	09	26	− 23	− 31	− 14
**Paraná**	1352	723	2089	1900	1045	2892	40	22	62	16	09	25	− 60	− 62	− 57
**Pernambuco**	1213	644	1851	1768	975	2709	33	18	50	19	10	28	− 43	− 48	− 38
**Piauí**	263	144	406	512	284	773	21	11	32	14	08	21	− 32	− 38	− 25
**Rio de Janeiro**	3369	1813	5170	3883	2127	5928	44	23	67	18	10	20	− 58	− 61	− 55
**Rio Grande do Norte**	313	173	479	552	308	838	20	11	31	15	08	22	− 28	− 35	− 19
**Rio Grande do Sul**	1860	998	2886	2269	1239	3463	37	20	57	15	08	24	− 58	− 60	− 55
**Rondônia**	78	41	121	203	110	314	34	18	53	16	09	25	− 51	− 57	− 45
**Roraima**	11	06	16	38	20	58	33	18	50	16	09	25	− 51	− 57	− 44
**São Paulo**	5757	3104	8882	7568	4155	11,619	38	20	59	16	09	24	− 60	− 62	− 57
**Santa Catarina**	655	346	1012	1001	552	1520	36	19	55	15	08	22	− 59	− 62	− 56
**Sergipe**	177	97	268	297	166	452	22	12	33	15	08	22	− 33	− 38	− 28
**Tocantins**	69	37	109	176	96	267	28	15	43	14	07	21	− 50	− 56	− 45

*U.I.* uncertainty interval

*Age-standardized rate (per 100,000 inhabitants)

†Breast cancer, colorectal cancer, diabetes mellitus, ischemic heart disease, and stroke

**Fig. 1:**
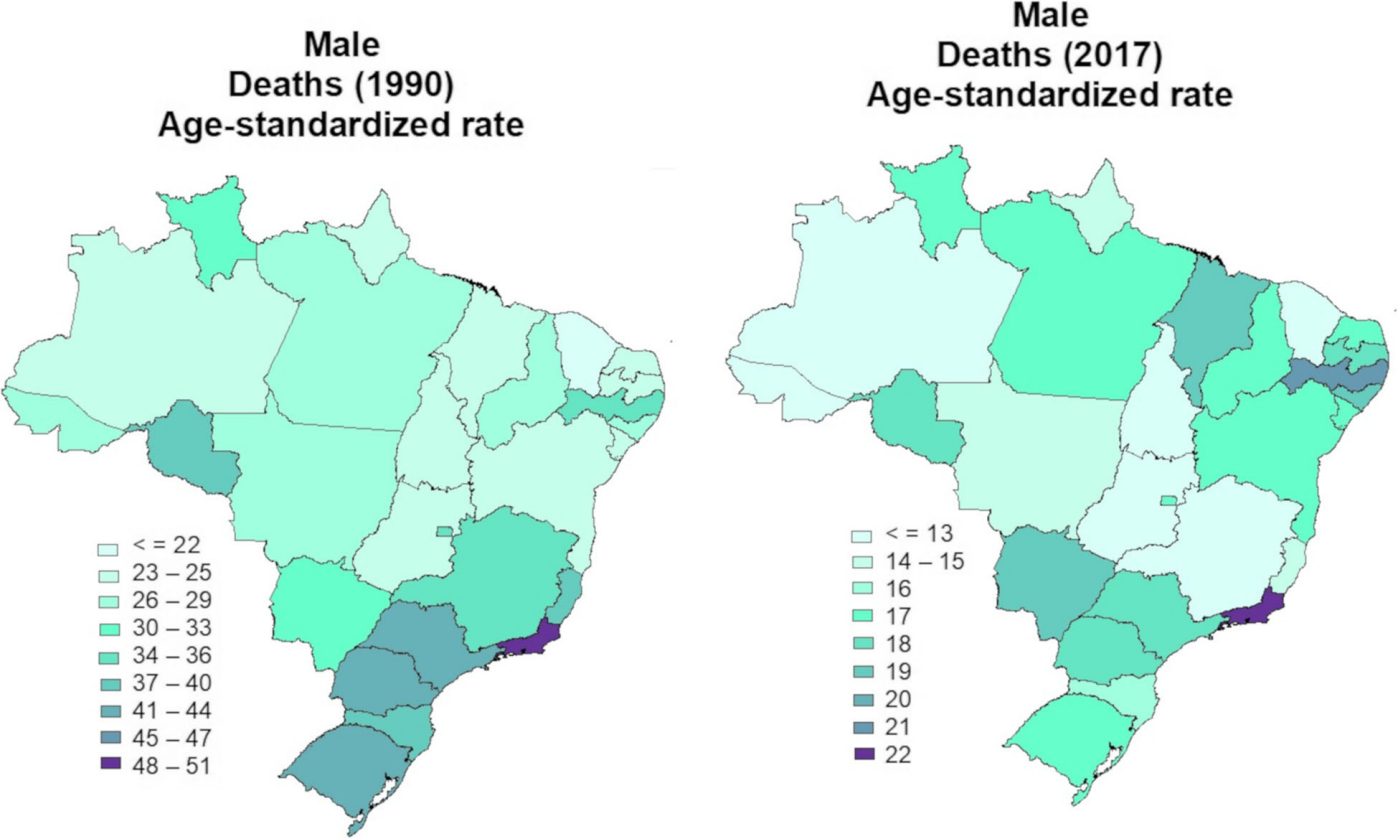
Age-standardized rate of deaths for all-causes attributable to physical inactivity in the Brazilian male population

The age-standardized mortality rate (per 100,000 population) for all causes attributable to physical inactivity in 1990 and 2017 in the Brazilian male population was 34 (95% U.I. 18–53) and 17 (95% U.I. 09–27), respectively. In 2017, there was a significant reduction compared to 1990 in age-standardized mortality rate for all causes attributable to physical inactivity in males from all Brazilian states. While in 1990, the highest age-standardized mortality rates for all causes attributable to physical inactivity were observed in men from the Southern and Southeastern states of Brazil; in 2017, the highest age-standardized mortality rates for all causes attributable to physical inactivity were observed in males from the North and Northeast of Brazil (Fig. 1).

**Fig. 2:**
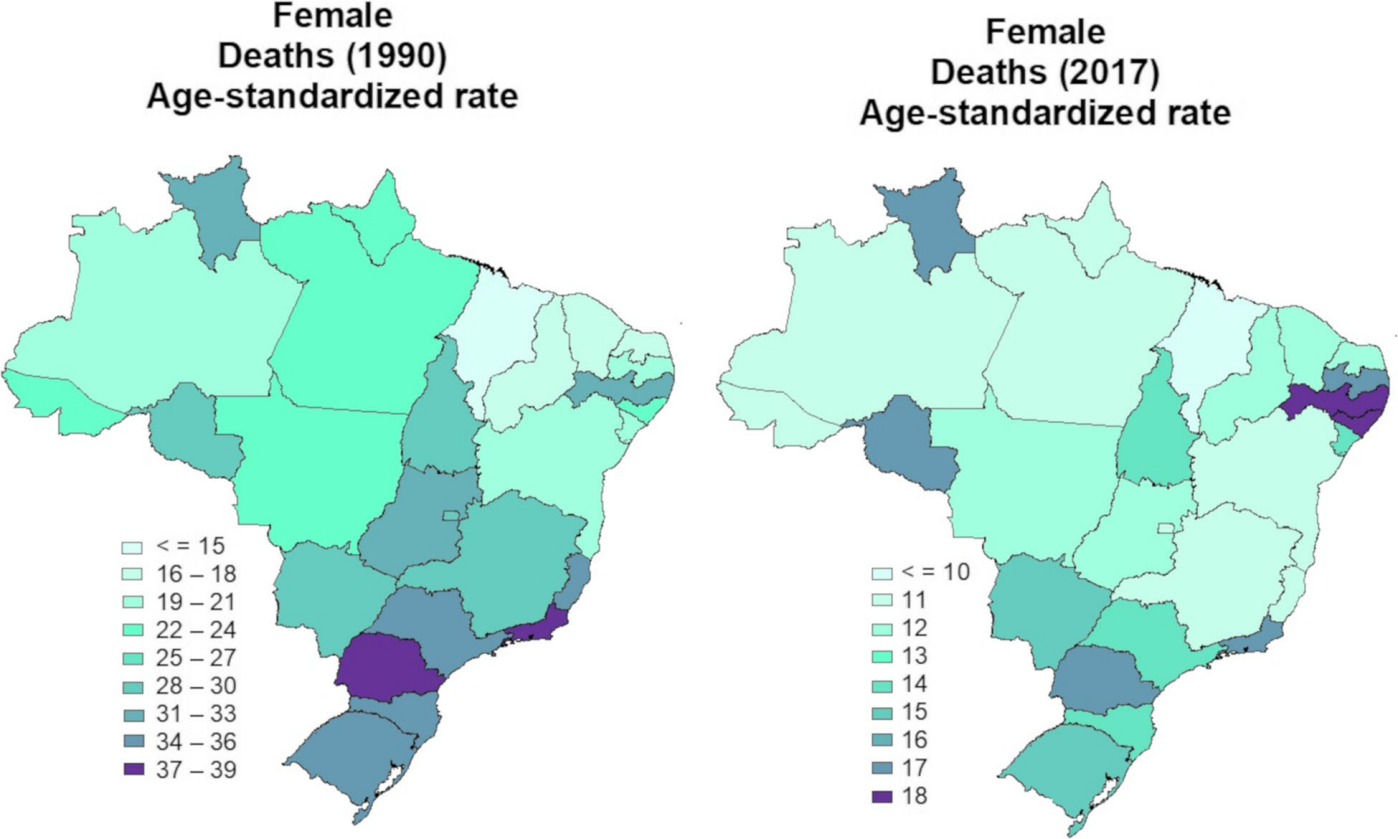
Age-standardized rate of deaths for all-causes attributable to physical inactivity in the Brazilian female population

The age-standardized mortality rate (per 100,000 population) for all causes attributable to physical inactivity in 1990 and 2017 in the Brazilian female population was 28 (95% U.I. 15–43) and 13 (95% U.I. 07–20), respectively. In 2017, there was a significant reduction compared to 1990 in age-standardized mortality rate for all causes attributable to physical inactivity in females from all Brazilian states. While in 1990, the highest age-standardized mortality rates for all causes attributable to physical inactivity were observed in females from the Midwest, Southern, and Southeastern states of Brazil; in 2017, the highest age-standardized mortality rates for all causes attributable to physical inactivity were observed in females from the Northeast of Brazil (Fig. 2).

The age-standardized mortality rates (per 100,000 inhabitants) for all causes attributable to physical inactivity increased as the age group of the Brazilian population increased in both sexes. In 1990, the PAF showed that approximately 1.0%, 3.0%, and 4.0% of all-cause deaths in males aged 25–49 years, 50–69 years, and ≥ 70 years, respectively, could be avoided if the Brazilian population reached 3000–4500 MET-min per week of physical activity. In 2017, the values were 0.6%, 2.4%, and 3.0% for males aged 25–49 years, 50–69 years, and ≥ 70 years, respectively. For females in 1990, the PAF showed that 1.3%, 3.5%, and 5.1% of all-cause deaths in aged 25–49 years, 50–69 years, and ≥ 70 years, respectively, could be avoided if the Brazilian population reached 3000–4500 MET-min per week of physical activity. In 2017, the values were 1.1%, 2.6%, and 3.3% for females aged 25–49 years, 50–69 years, and ≥ 70 years, respectively (Table 2).

**Table 2:** Age-standardized mortality rate and the population attributable fraction in Brazilian population

	Mortality due to all-causes^**†**^ attributable to physical inactivity			
	Male		Female	
	Rate* (95% U.I.)	PAF: % (95% U.I.)	Rate* (95% U.I.)	PAF: % (95% U.I.)
**1990**				
**25–49 years**	3.2 (1.6–5.1)	0.8 (0.4–1.4)	2.0 (1.0–2.8)	1.3 (0.7–2.0)
**50–69 years**	58.8 (31.4–92.6)	3.2 (1.7–5.0)	37.0 (20.2–56.7)	3.5 (1.9–5.3)
≧ **70 years**	311.7 (166.7–480.6)	4.1 (2.2–6.4)	310.2 (167.8–473.8)	5.1 (2.8–7.8)
**2017**				
**25–49 years**	1.9 (0.9–3.1)	0.6 (0.3–1.0)	1.2 (0.6–1.9)	1.1 (0.6–1.7)
**50–69 years**	32.3 (17.2–50.7)	2.4 (1.3–3.8)	19.6 (10.9–29.7)	2.6 (1.4–3.9)
≧ **70 years**	183.8 (101.9–282.3)	3.0 (1.7–3.8)	166.3 (92.6–251.5)	3.3 (1.8–5.0)

*PAF* population attributable fraction, *U.I.* uncertainty interval

*Rate per 100.000 inhabitants

†Breast cancer, colorectal cancer, diabetes mellitus, ischemic heart disease, and stroke

**Fig. 3:**
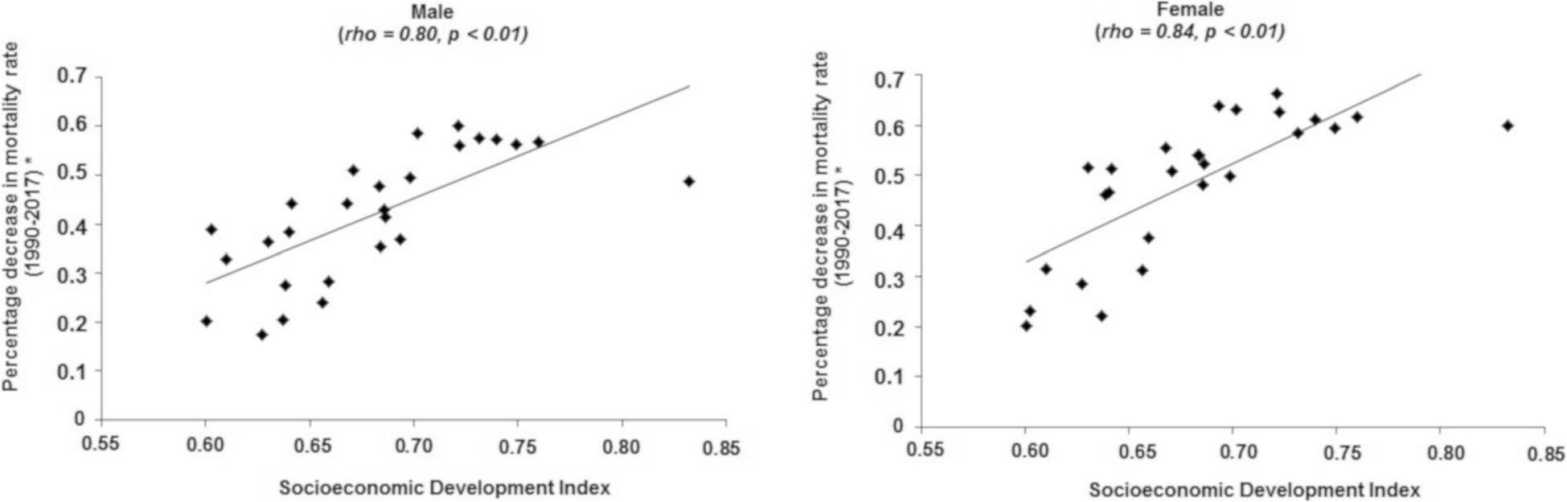
Relationship between percentage decrease in age-standardized rate of deaths due to physical inactivity and SDI. Note, age-standardized rate; rho, Spearman’s correlation coefficient

Figure 3 shows the relationship between the decreases in age-standardized mortality rate for all causes attributable to physical inactivity with the SDI of the Brazilian states. The Brazilian states with better socioeconomic conditions presented greater reductions in age-standardized mortality for all-causes due to physical inactivity (male: rho = 0.80; female: rho 0.84) rates over the period of 28 years.

## Discussion

In this study, from 1990 to 2017, a decrease in standardized death rate from all causes attributable to physical inactivity was observed in Brazil. This result demonstrates Brazil’s progress in this scenario. At least three hypotheses could be given to explain these results. The first hypothesis is that the Brazilian population is more physically active compared to the 1990s, and therefore, there was a decrease in standardized death rate from all causes attributable to physical inactivity. To illustrate this hypothesis, a survey conducted from 2006 to 2016 in all states of Brazil aimed to analyze time trends in leisure-time physical activity in adults [17]. The authors found that there was an increase both in the percentage of leisure-time physical activity (from 44.0 to 53.6% or 0.97 percentage points per year) and in the percentage of individuals that achieved recommended levels of physical activity (i.e., ≥ 150 min/week), from 30.3 to 37.6% (1.20 percentage points per year) [17]. However, this study cited found this increase considering physical activity during leisure time.

This study showed an important metric in global health estimates (i.e., SEV) that does not fully accept the first hypothesis. Information about SEV revealed that the risk of the Brazilian population have been exposed to physical inactivity was stable over 28 years (SEV = 59% in 1990, and SEV = 59% in 2017). One of the factors that justify this stability is the urban and technological development that Brazil has undergone during these 28 years, resulting in less involvement in daily physical activities and greater propensity for physically inactive lifestyles [7, 18].

The second hypotheses that could explain the reduction in age-standardized mortality rates for all causes attributable to physical inactivity from 1990 to 2017 in Brazil are the successful actions for prevention, diagnosis, and treatment of chronic noncommunicable diseases in Brazil over almost three decades. A research conducted in all states of Brazil aimed to analyze the mortality trends for chronic noncommunicable diseases in the period 2000–2013 and its probability of death until 2025 [19]. The authors found that there was an average decline of 2.5% per year in all four major chronic noncommunicable diseases in Brazil (i.e., cardiovascular diseases; respiratory diseases; malignant neoplasms; diabetes mellitus), and the probability of premature death was reduced from 30% in 2000 to 26.1% in 2013, and it was estimated that this probability reduces to 20.5% in 2025 [19]. In addition, Brazil has well-established policies to combat chronic noncommunicable diseases since 2011 [20].

The second hypothesis presented above may not be fully accepted if one analyzes the trends in age-standardized mortality rate from breast cancer, colorectal cancer, ischemic heart disease, diabetes mellitus, and stroke in Brazil. These five causes of mortality were those studied in the present study as attributable to physical inactivity. The study of breast cancer mortality trends revealed that there was stability in age-standardized mortality rate from 1990 to 2015 in Brazilian women [21]. For colorectal cancer, there was an increased in age-standardized mortality rate from 1990 to 2015 in men in Brazil, and there was a stability in women [21]. For stroke [22], ischemic heart disease [23], and diabetes mellitus [24], there was decreased in age-standardized mortality rate from 1990 to 2015 in men and women in Brazil.

The third hypothesis to explain the reduction in age-standardized mortality rates for all causes observed in the present study and that seems to explain, in part, these results is the fact that Brazil has been successful in reducing other risk factors for noncommunicable chronic diseases [25, 26]. The prevalence of smokers in Brazil dropped 0.65 points percentage per year from 2006 (15.6%) to 2014 (10.8%) [25]. In addition, from 2008 to 2015, there was an increase in the consumption of fruits and vegetables in the Brazilian population [26]. These risk factors are also related to the diseases investigated in the present study (i.e., stroke, breast cancer, colorectal cancer, ischemic heart disease, and diabetes mellitus), and this can interfere with the estimation models of mortality.

Thus, it seems to be more cautious to state that a combination of the first, second, and third hypothesis presented justifies the findings of the present study. For, while at the same time that actions of prevention, diagnosis, and treatment of chronic noncommunicable diseases in Brazil were implemented [20], policies of promotion of the physical activity were implemented [27]. These efforts are needed to decrease mortality by noncommunicable chronic diseases.

The present study found that from 1990 to 2017, Brazilian states with the lowest SDI and with the greatest social discrepancies had lower reductions in age-standardized mortality rates for all causes due to physical inactivity when compared to states with better economic development. This finding reinforces two problems that have already been presented in literature [18, 28]: (1) physical inactivity is a serious problem in economically disadvantaged populations; (2) social and economic inequalities present in these states are reflecting on the health of the population, causing greater difficulty in reducing physical inactivity. Thus, increasing the level of physical activity of the population in order to reduce the risks of early mortality and morbidity due to noncommunicable chronic diseases should include efforts to improve the living conditions and reduce inequities of the population and not only isolated physical activity promotion actions.

This study corroborates what was demonstrated in a previous study [1] in which mortality due to physical inactivity was greater as the age group increases. The aging process alone leads to an increased risk of chronic diseases and noncommunicable chronic diseases and a reduction in daily physical activities [1, 7, 18], which makes it clear that initiatives to promote active and healthy aging should be prominent in Brazil.

The fact that this study considers only the outcome information on stroke, breast cancer, colorectal cancer, ischemic heart disease, and diabetes mellitus is a limitation. Although there is consistent evidence that physical inactivity is an independent risk factor for these diseases [3], there is also some evidence for other health problems and chronic diseases [29], which were not considered in the present study. Because of this, it is likely that the burden of disease associated with physical inactivity is underestimated. The other limitation of this study includes the measurement of physical activity through different questionnaires that although result in an estimate of the level of physical activity, can also result in error and bias resulting from different forms of interpretation, different questionnaires, and possibly social desirable responses. In addition, a questionnaire is a subjective measure of physical activity. The other limitation of this study is the non-stratification of physical activity by different domains so that a deeper knowledge of the different physical activity patterns would be presented. Another limitation evident in this research is that the 95% U.I. for the age-standardized SEV is very large, and therefore, it is difficult to draw conclusions on this.

## Conclusions

It could be concluded that physical inactivity contributed to a substantial number of deaths in Brazil and in the different Brazilian states from 1990 to 2017. From 1990 to 2017, a decrease in standardized death rate from all causes attributable to physical inactivity was observed in Brazil. Brazilian states with the highest social inequalities showed lower reductions (from 1990 to 2017) in age-standardized mortality rate for all causes attributable to physical inactivity. The results of the present study show the importance of preventing risk factors for noncommunicable chronic diseases in all Brazilian states, and greater effort in combating social and economic inequities related to the living conditions of the population is needed, so that the adoption of active and healthy lifestyle has greater reach in all regions of Brazil.

## Data Availability

Data we used in this article are publicly available online on the official website of Institute of Health Metrics and Evaluation (http://ghdx.healthdata.org/gbd-results-tool).

## Abbreviations

DALYs: Disability-adjusted life-years
GBD: Global Burden of Disease
GPAQ: Global Physical Activity Questionnaire
IHME: Institute for Health Metrics and Evaluation
IPAQ: International Physical Activity Questionnaire
PAF: Population attributable fraction
SDI: Socioeconomic Development Index
SEV: Summary exposure value
ST-GPR: Spatiotemporal Gaussian process regression
TMREL: Theoretical minimum-risk exposure level
U.I.: Uncertainty intervals
